# Acoustic imaging of stable double diffusion in the Madeira abyssal plain

**DOI:** 10.1038/s41598-024-58861-7

**Published:** 2024-04-09

**Authors:** Ana F. Duarte, Álvaro Peliz, Luís Matias, Renato Mendes, Leonardo Azevedo

**Affiliations:** 1 CERENA/DER, Instituto Superior Técnico (IST), Universidade de Lisboa, Av. Rovisco Pais 1, 1049-001 Lisbon, Portugal; 2 Instituto Dom Luiz (IDL), Faculdade de Ciências, Universidade de Lisboa, Campo Grande, 1749-016 Lisbon, Portugal; 3 +ATLANTIC CoLAB, Museu das Comunicações, Rua do Instituto Industrial 16, 1200-225 Lisbon, Portugal; 4 Underwater Systems and Technology Laboratory (LSTS), Associate Laboratory for Energy, Transports and Aerospace (LAETA), Faculdade de Engenharia, Universidade do Porto (FEUP), Porto, Portugal

**Keywords:** Physical oceanography, Geophysics

## Abstract

Sub-mesoscale and mesoscale (i.e., 1–10 km and 10–200 km, respectively) ocean processes are highly relevant for the understanding of global circulation, mixing of water masses and energy exchange between ocean layers. However, the processes happening at these scales are hard to be characterized using direct measurements of temperature and salinity. Direct measurements are obtained from vertical probes and/or autonomous vehicles, which, despite their high vertical resolution, are sparsely located in space and therefore unable to capture spatial details at these scales. Seismic oceanography (SO) data have been successfully used to imaging and characterize the ocean at these spatial scales. These data represent indirect measurements of the ocean temperature and salinity along kilometric transects with high horizontal resolution (i.e., a near-synaptic view of the system under investigation), but lower vertical resolution when compared with direct observations. Despite its complex oceanographic setting, the Madeira Abyssal Plain is still largely uncharacterized due to the lack of direct observations. We show for the first time a comprehensive processing, modelling and interpretation of three 2-D seismic oceanography sections from this region. The data show coherent seismic reflection in space, depth and time and shed light into this oceanographic setting with an unprecedent horizontal resolution. The SO modelling and interpretation are combined with existing direct measurements and a quantitative method to correlate thermohaline staircases interpreted from conductivity-temperature-depth casts and seismic reflections is proposed. The results show the relatively stable presence of thermohaline staircases in simultaneously time and space between 1200 and 2000 m of water depth and their spatial variability and contribute to the generalization of SO in physical oceanography.

## Introduction

**Figure 1:**
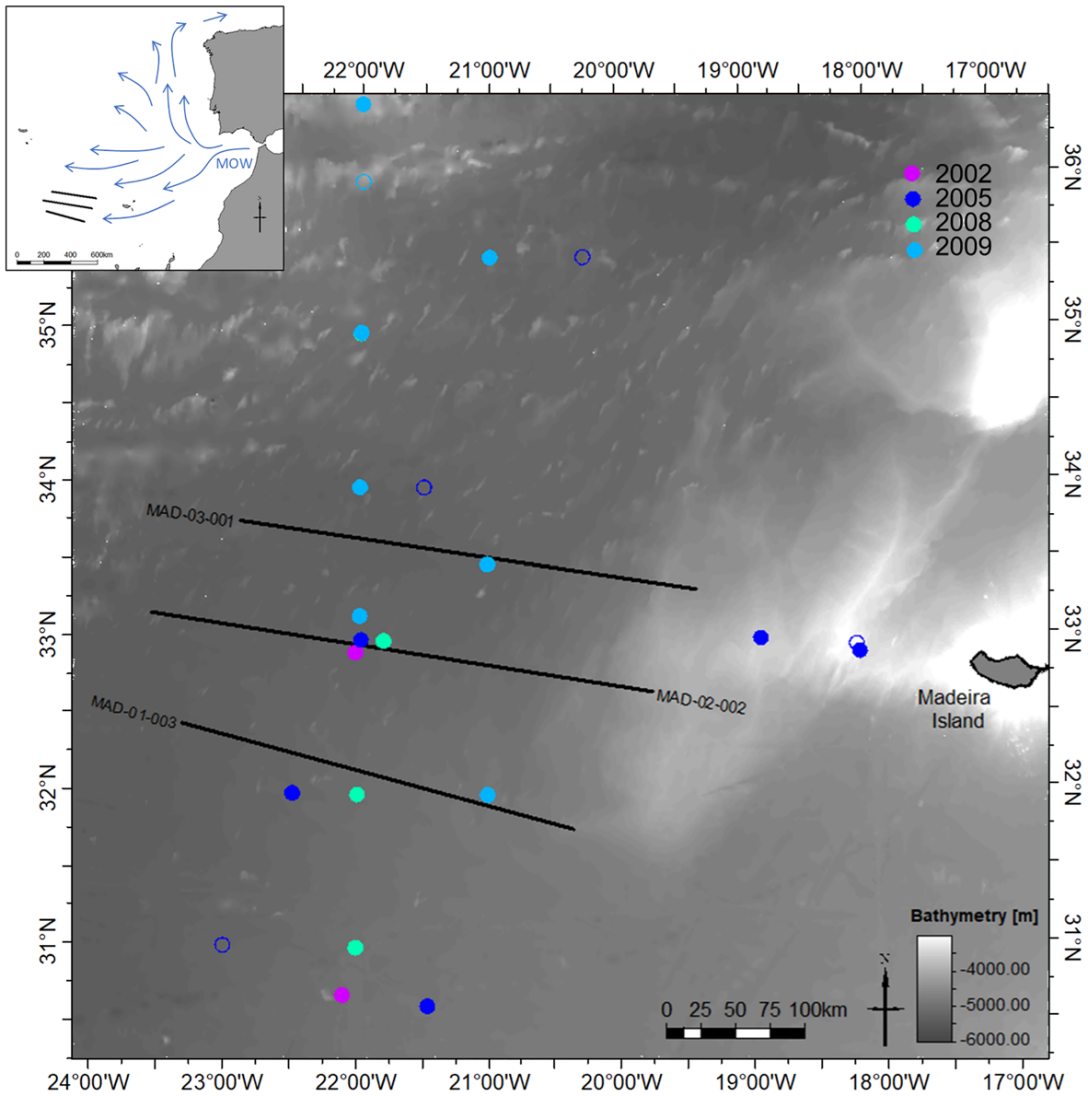
Location of the available data set. The black lines represent 2-D multichannel seismic reflection sections, the CTD locations are represented by circles where each year is plotted with a different color: 2002 (purple), 2005 (dark blue), 2008 (green), 2009 (light blue), filled circles represent CTDs with the presence of thermohaline staircases.

The Mediterranean outflow water (MOW) highly influences the thermocline structure of the Madeira Abyssal Plain (MAP) (Fig. 1). The MOW is characterized by warm and high-salinity water masses, and in this region can be identified through direct measurements of the ocean water temperature and salinity between ~ 500 to 1500 m of water depth^1^. The MOW creates a temperature and salinity gradient anomaly dividing the North Atlantic Water (NAW) from the North Atlantic Deep Water (NADW). This salinity gradient anomaly is auspicious for developing double-diffusive mixing^2^, such as thermohaline staircases formed by mixing of the MOW with NADW. This phenomenon is known to happen in the MAP region between ~ 1600 to 2000 m water deep^3^. Double-diffusive mixing, translated into salt fingering and diffusive convection, occurs in the ocean when the gradient of temperature or salinity is unstable^4^. In the MAP is mainly identified from direct measurements of the water's conductivity and temperature as a function of depth. These measurements are acquired along vertical profiles using conductivity-temperature-depth (CDT) casts. Despite the high vertical resolution of these measurements, they are spatially sparse and unable to perform a detailed characterization of the spatial extent and lateral variation of these processes at the ocean sub-mesoscale and mesoscale (i.e., 1–10 km and 10–200 km, respectively).

Vertical ocean temperature and salinity profiles analyzed in Azevedo et al.^5^, van der Boog et al.^6^ and You^7^ suggest the presence of double diffusion features, such as thermohaline staircases, in the MAP region. Thermohaline staircases are associated with small-scale double-diffusive mixing that convert the quasi-continuous vertical temperature and salinity profiles into step-like layered structures consisting of abrupt changes in temperature and salinity, separating regions where the temperature and salinity fields are relatively constant^4,8,9^. This phenomenon globally has a small contribution to the energy budget, however regionally it increases the diapycnal mixing rates, controlling nutrient fluxes and possibly affecting the Ocean Meridional Overturning^6,10,11^. Observations of thermohaline staircases in the Arctic^12^, in the Tyrrhenian Sea^13,14^ and in the classic experiments of the Caribbean Sea^15^, show individual layers over scales from few meters to up to hundreds of meters, respectively, and that can often demonstrate long-term persistence, lasting from months to years, and exhibit strong lateral coherence, spanning distances ranging from several tens of kilometers to hundreds of kilometers. Although CTD data provide good-quality profiles with high vertical resolution, its measurements are only taken at discrete locations, making it challenging to map thermohaline staircases spatially and their lateral variations. As a complementary source of information, the ocean's vertical structure, and associated physical properties, may be studied using acoustic data^12^.

Seismic oceanography (SO)^16^ uses multichannel reflection data (MCS) to imaging the water column structure along two-dimensional sections and, recently, in three-dimensional volumes^17^. The recorded acoustic response depends on variations in ocean temperature and salinity in the water. In seismic oceanography, acoustic sources generate energy that is propagated in the water column. These wave fields are reflected (i.e., weak reflections) back to the surface at interfaces between water masses with different temperature and salinity and recorded in hydrophones near the sea surface^18^. Seismic reflections happen at abrupt changes in temperature and salinity, having temperature variations contributing with ~ 80% for the reflection coefficient^18,19^. After processing, and depending on the acquisition setting, these data might have a vertical resolution of a few of meters. The vertical resolution of seismic oceanography data is lower than CTD profiles but have a horizontal resolution of ~ 6 to ~ 25 m depending on the data acquisition geometry. Depending on the oceanographic setting where these data are acquired, the processed SO images might show continuous interfaces between those thermohaline layers, both laterally and in depth^14^. SO data represent a complementary source of information to traditional oceanographic casts and provide the possibility of obtaining ocean temperature and salinity models in high-resolution and to interpolate those characteristics in three-dimensions.

The qualitative interpretation of SO sections might provide valuable oceanographic insights to understand mixing processes and phenomena occurring at different water column depths^20^. The advantage of this method is the capability of producing images of the ocean with horizontal and vertical resolutions of few meters throughout long distances, usually kilometers, providing a near-synoptic view of the ocean and potentially enabling the study of fine-scale ocean processes, as the example of the thermohaline staircases, eddies formed by the MOW and thermohaline fronts^16,18,21,22^. Buffett et al.^14^ have imaged thermohaline staircases, ranging from 50 to 500 m in thickness, with SO data in the Tyrrhenian basin, Central Mediterranean Sea. They additionally show the ability of these data to visualize multiple interfaces within each thermohaline staircase. However, a quantitative correlation method to couple thermohaline staircases identified in SO and oceanographic data has not been proposed.

We apply herein SO to imaging and model the space, depth, and time structure of the ocean meso- and sub-mesoscale of the MAP region. We focus on the modelling and interpretation of parallel and coherent seismic reflections at approximately 1500 m water depth, using three parallel 2-D multichannel seismic reflection sections, which were quantitatively correlated to thermohaline staircases identified in CTD profiles acquired within the study area^14,20^.

### Data set description

#### Seismic oceanography data

We used a set of three parallel 2-D multichannel seismic reflection sections acquired by the Portuguese Task Force for the Extension of the Continental Shelf in the MAP to the west of Madeira Island (Fig. 1). Each section is approximately 300 km long along the East–West direction and are approximately 100 km apart from each other. The acquisition of these data started on June 6, 2006 and ended on June 12, 2006. The 2-D sections were acquired chronologically from north to south.

The multichannel seismic reflection data were recorded with a sampling interval of 2 ms. The source was fired every 50 m, and the signal was recorded by receiver groups located 12.5 m apart from each other while the acquisition vessel sailed at approximately 4 knots. The acquisition geometry determines the spatial resolution of the images obtained after the data processing and this acquisition geometry results in a common reflection midpoint every 6.25 m. The dominant frequency of the data is about 50 Hz, resulting in a vertical resolution of approximately 15 m, while the horizontal spatial resolution is close to the spacing between common midpoint reflections (i.e., 6.25 m). Although the vertical resolution is smaller than the one obtained from conventional oceanographic casts, the horizontal resolution is higher than that obtained from ordinary oceanographic measures, allowing a detailed spatial analysis of the ocean phenomena.

**Figure 2:**
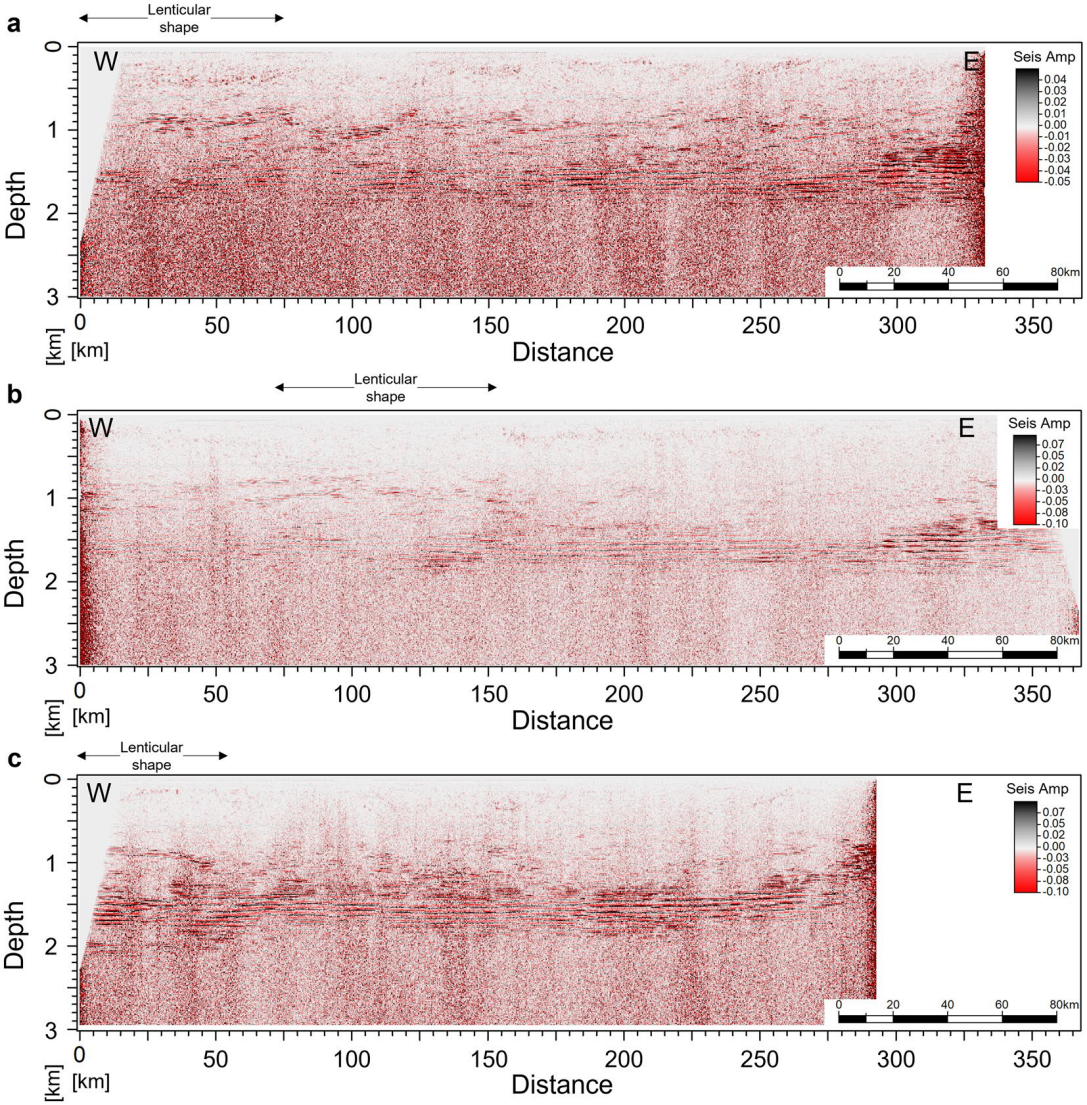
Processed 2-D seismic sections from Madeira abyssal plain from the sea surface down to 3 km depth: (**a**) MAD-03-001, (**b**) MAD-02-002, (**c**) MAD-01-003. For location of the sections, see Fig. 1.

We focus our modelling and interpretation of the SO data to the upper part of the sections, down to 2 km depth, where the key and spatially coherent seismic reflections are present (Fig. 2). In general, for the three 2-D SO sections, from the sea surface until ~ 800 m of water depth, the reflections have a low amplitude content and are spatially discontinuous along the horizontal direction (Fig. 2). This effect might be related to artifacts produced due to the attenuation of the direct wave during data processing and because the ocean water at the spatial resolution of the SO data is homogeneous. Below the ~ 800 m of water depth (Fig. 2), the amplitude content increases, suggesting more heterogeneous and variable water mixing processes, both horizontally and vertically. At these depths, we also expect to find the influence of the MOW. The seismic oceanography signature of the influence of the MOW can be observed by relatively spatially coherent seismic reflections forming a circular–lenticular shape, as highlighted in Fig. 2 between 1000 and 1500 m of water depth. These features have similarities to eddies originating due to the MOW (i.e., Meddies) investigated by Biescas et al.^21^ in the Gulf of Cadiz and Iberia Margin. Between 1500 and 2000 m of water depth, the sections show a seismic signature with parallel and continuous horizontal reflections. These are present in almost all the extent of the 2-D SO sections, and the interface can be traced as a single reflection up to ~ 200 km. Due to the spatial coherency of the continuous horizontal reflections in the three SO sections, we focus our quantitative interpretation in these events and assess the possibility of these reflections being originated due to thermohaline staircases. To support the interpretation of these seismic reflections, and correlated them with true oceanographic events, we first analyze the available direct measurements (i.e., the CTD data) to assess whether those horizontal reflections indicate the presence of thermohaline staircases.

### Direct ocean measurements

We used the temperature and salinity profiles from CTD probes for two complementary reasons. First, to validate the indirect information provided by the set of 2-D multichannel seismic reflection sections. Second, to support the analysis of the seismic reflections potentially associated with thermohaline staircases. The CTD data were acquired in 2002, 2005, 2008 and 2009 by R/V Poseidon during cruises POS283, POS321, POS377 and POS383, respectively^23–26^. These data cover the spatiotemporal distribution of the 2-D multichannel seismic reflection sections (Fig. 1) capturing the water column heterogeneities down to 2000 m (Fig. 2). From the original CTD data set, we considered the 23 profiles inside our study area (i.e., within the box defined by 36° 37′ N 25° 01′ W and 30° 11′ N 16° 30′) (Fig. 1). Each measurement reached 2000 m in depth. From this dataset, we gathered the 18 CTDs that exhibit clear evidence of thermohaline staircases in temperature and salinity profiles (location on Fig. 1). When identifying thermohaline staircases in the CTD data, CTDs with less than two interpretable steps were discarded as we opted for a conservative approach to reduce the uncertainty degree associated with the interpretation of the CTD data.

## Results

### Qualitative interpretation of the direct measurements (CTDs)

**Figure 3:**
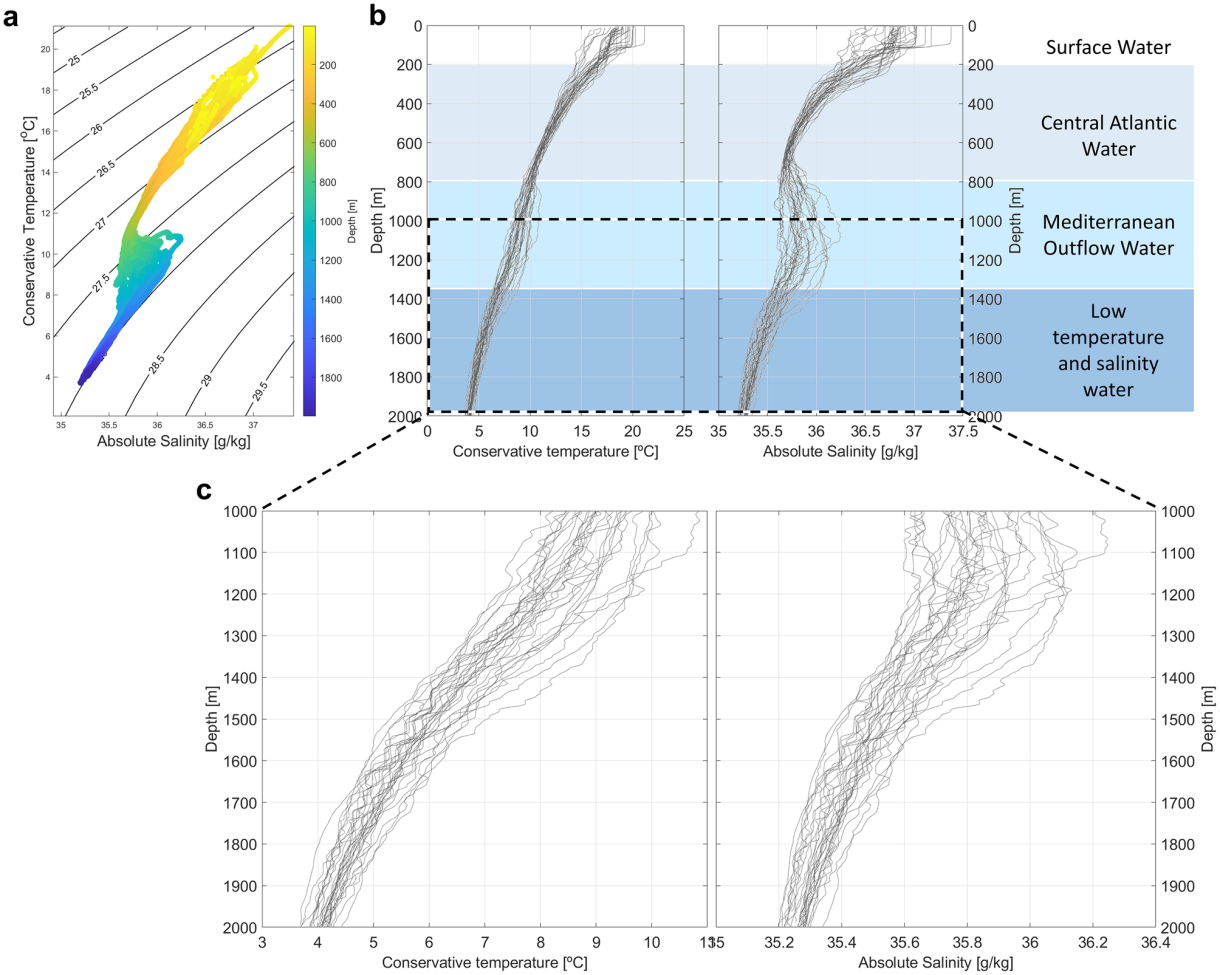
CTD measurements of temperature and salinity for the eighteen locations considered. (**a**) CT and SA diagram colored as a function of depth, with potential density contours overlain. (**b**) CT and SA profiles through depth and description of corresponding water masses for the water column. (**c**) Detail of the CT and SA profiles to emphasize the step-like features observed from 1000 to 2000 m.

The typical vertical structure of the subtropical Northeast Atlantic is observed in the conservative temperature (CT) and absolute salinity (SA) diagram (Fig. 3a) calculated from the CTD profiles. We can roughly classify four main water masses layers (Fig. 3b): Surface water, Central Water (here the Eastern North Atlantic Central Water), the warmer and higher salinity Mediterranean Outflow Water (MOW ~ 500 to 1500 m intermediate layer), below which we find colder and fresher transformed water masses that result from mixing of intermediate and deep waters originating from the Arctic and Antarctic^27^. The MOW contrasts significantly in temperature and salinity with the layers above and below that is translated in a s-shaped TS-diagrams for this region (Fig. 3a). From 1000 until 2000 m, most of the CTD profiles exhibit step-like features, typical of thermohaline staircases (Fig. 3c).

**Figure 4:**
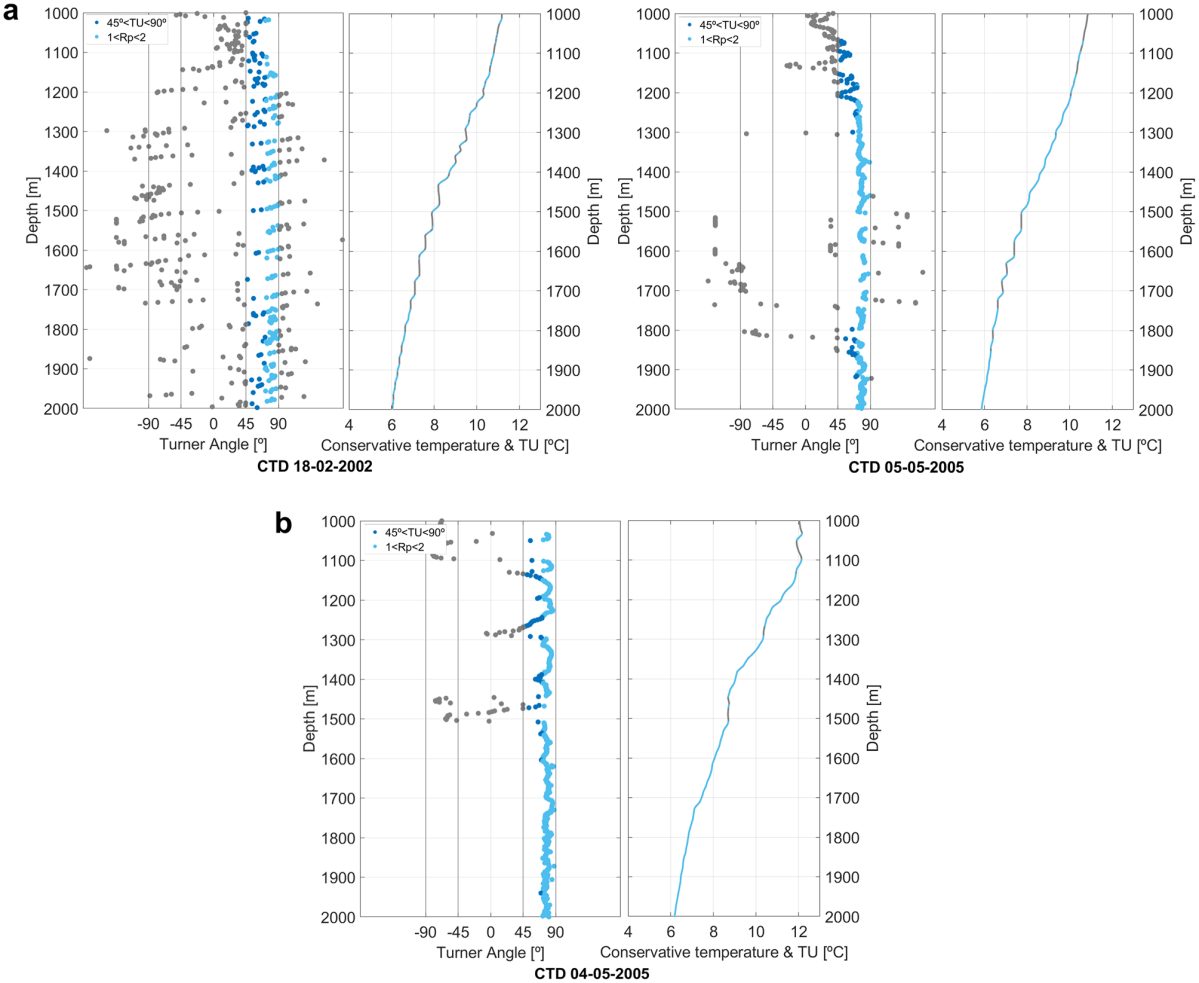
TU and CT profiles. Defining salt finger-prone zones for two CTDs with: (**a**) well defined thermohaline staircases; and (**b**) with lack of definition. These plots were calculated from the moving average of 7 points for CT and SA to minimize noise.

We focus on the interpretation of the CTD in those depths. To test the effective presence of thermohaline staircases, we analyze the Turner angle ($TU$) and the density ratio ($R\rho$) (Eq. 5), parameters that, when combined, are helpful to determine the origin and intensity of the double-diffusive regime ($TU$ and $R\rho$ are described in detail in the Methodology section). Thermohaline staircases are defined as the regions in the CTD casts where $TU$ s range between 45° and 90° and $R\rho$ s between 1 and 2. Figure 4 shows two examples of CTD profiles where thermohaline staircases are clearly identified (Fig. 4a), and one example of a CTD cast without well-defined thermohaline staircases (Fig. 4b). When $TU$ and $R\rho$ conditions are favorable for the generation of thermohaline staircases, the corresponding CTD samples are highlighted and colored in blue (Fig. 4a). It is easily perceived that the salt finger prone layers correspond to the horizontal part of the staircases, where the mixing of heat and salt happens, followed by a layer of well-mixed water, with temperature and salinity constant through depth. In Fig. 4b, although it is possible to interpret the presence of a salt finger regime due to favorable $TU$ and $R\rho$ conditions, the step-like structures originated from the contrast of layers with constant temperature is not present in the conservative temperature profile, leading to a profile without a well-defined staircase.

These calculations allow inferring the presence of thermohaline staircases in the CTD casts, further results were obtained considering only 18 of all the CTD casts, where thermohaline staircases were clearly identified. These features are compared against the observed seismic reflections in the 2-D SO sections to validate the origin of the observed seismic reflections. First, we calculate the synthetic seismic response of the CTD profiles to compare numerically simulated seismic data with the observed seismic reflections. Then, we estimate the depth and spatial variability of the phenomenon along the 2-D SO sections.

**Figure 5:**
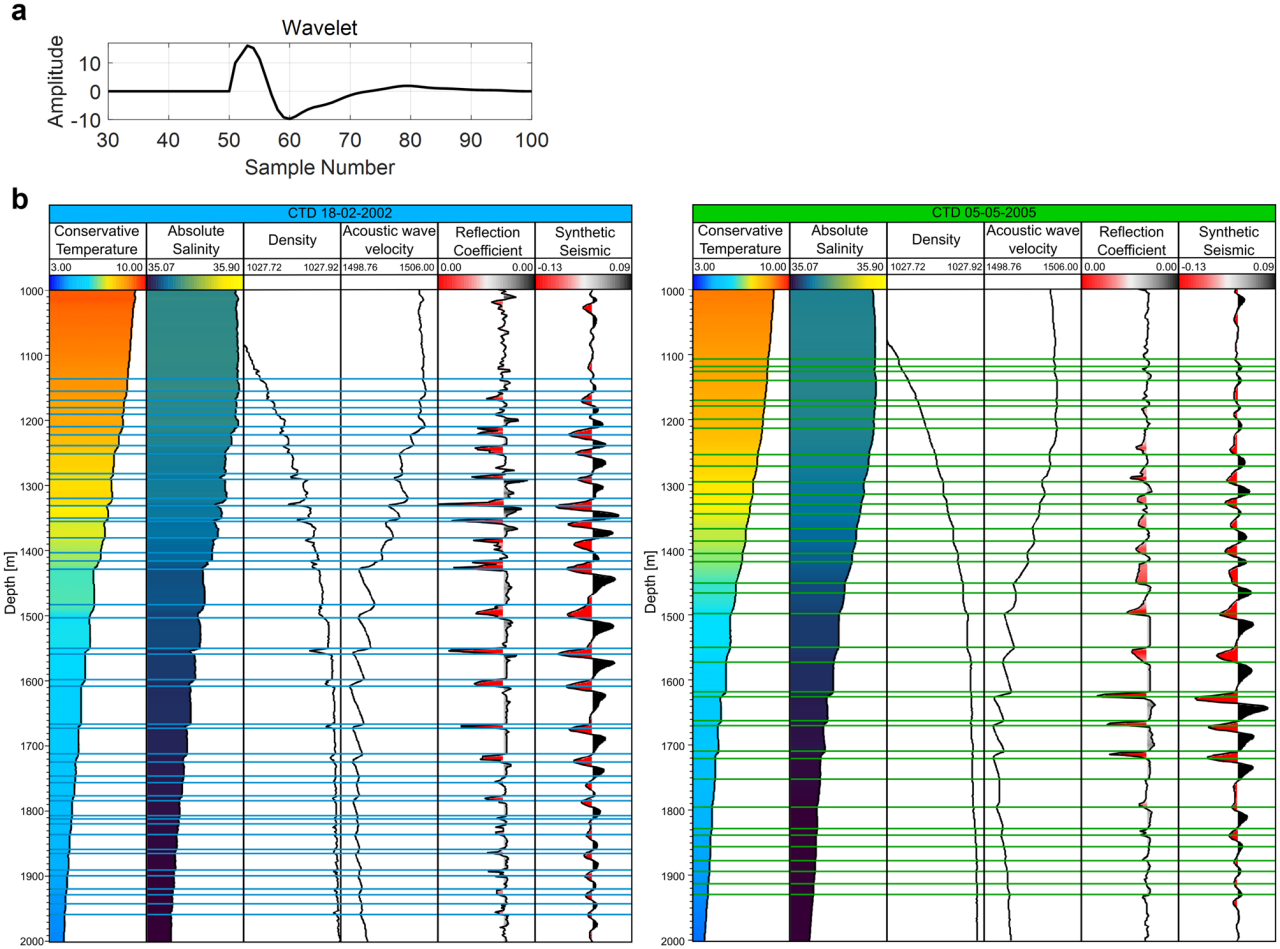
Seismic response calculated from the CT and SA measurements. (**a**) Wavelet extracted from the seismic section MAD-01-003; (**b**) Transformation of CT and SA into the synthetic seismic response. Profiles of two CTDs represented as blue (left) and green (right), Tracks from left to right for each CTD represent: CT; SA; density; sound speed; reflection coefficient; and synthetic seismic trace. Tops and bases of each thermohaline staircase were picked manually from the CT profile (blue and green lines corresponding to each CTD), starting at 1000 m and ending at 2000 m.

To compare both information from the CTD and the seismic reflections, we used the TEOS-2010^28^ to compute the corresponding reflection coefficients, which were then convolved with a wavelet extracted from the 2-D SO sections using a statistical method^29^ (Fig. 5). The wavelet aims to bring the synthetic calculations into the vertical scale of the SO data. The maximum negative amplitudes (i.e., seismic troughs and negative reflection coefficients) correspond to the depth of the salt finger layers of the thermohaline staircases. The layers of constant CT and AS correspond to the maximum amplitudes (i.e., seismic peaks and positive reflection coefficients).

### Joint interpretation of SO and CTD data

**Figure 6:**
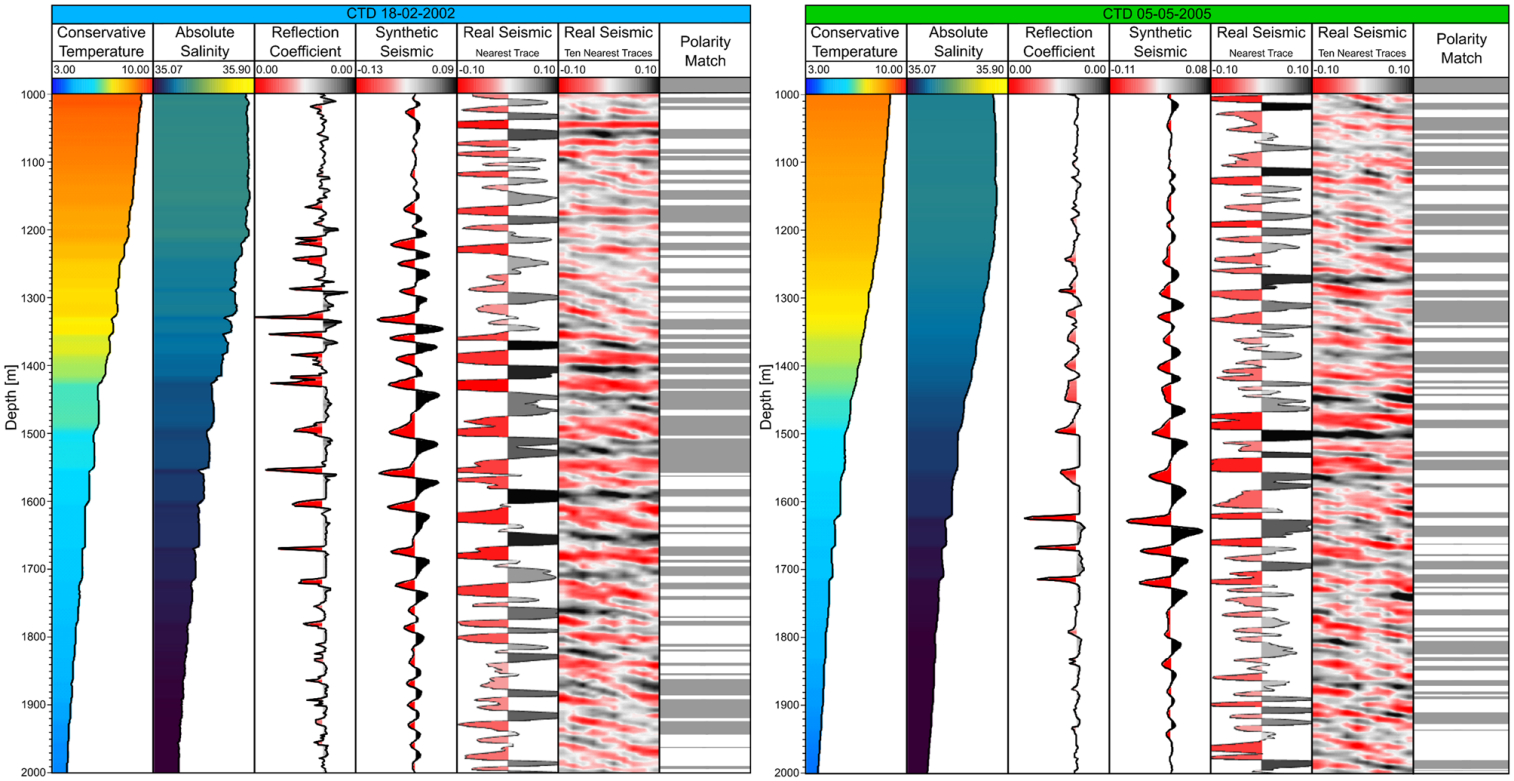
Comparison of the synthetic seismic response from CT and SA with the real seismic traces. Profiles of two CTDs represented as blue (left) and green (right). Tracks from left to right for each CTD: CT; SA; reflection coefficient; synthetic seismic trace; closest observed seismic trace; ten closest observed seismic traces; and polarity match.

We performed the joint interpretation of both kinds of data qualitatively and quantitatively. We first compare the synthetic SO response from the CTD data against the observed SO seismic trace with the smallest distance to that CTD profile (Fig. 6). Even though the seismic sections (acquired in 2006) are not contemporaneous nor collocated with the direct measurements from 2002 and 2005, distancing from the southern section ~ 160 and ~ 30 km, respectively, we find a good match between both synthetic and real seismic data. These results imply that the thermohaline staircases phenomenon observed in the MAP region is stable simultaneously in space, depth and time.

As a first approach before deepening further the analysis between the seismic sections with the synthetic SO response from the CTDs, we assess the fit between synthetic and observed seismic traces visually and through the polarity match between both data (i.e., signals with the same polarity). The polarity match is 61% for CTD 18-02-02 and 55% for CTD 05-05-05, represented as gray areas in Fig. 6 for each CTD. We should take into consideration that since the seismic sections are not contemporaneous nor collocated of the CTD casts, there is the possibility of some phase shifts between both data, resulting in lower polarity matches.

**Figure 7:**
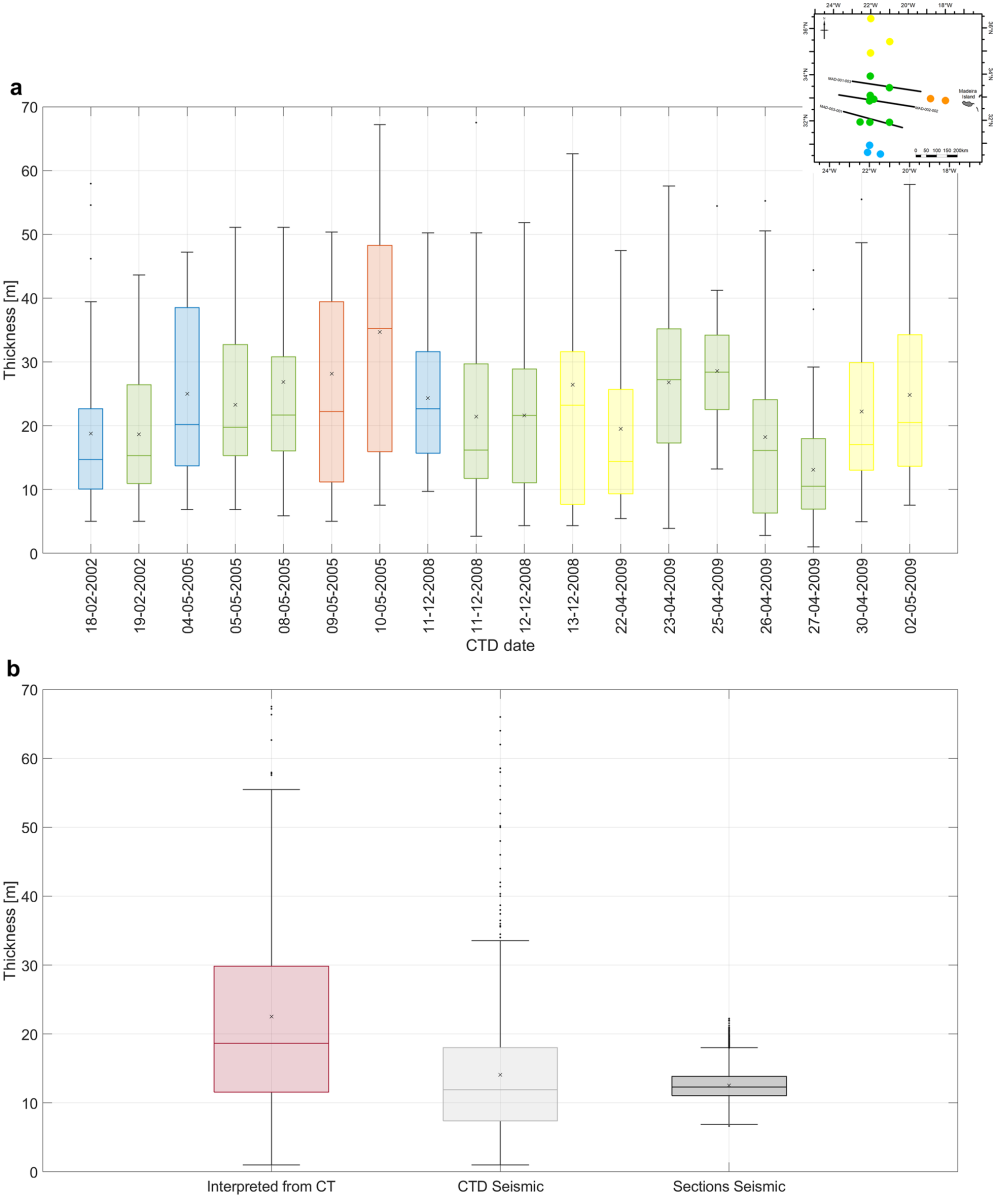
Thickness of the thermohaline staircases picked manually from the CT and SA profiles and calculated by the SO sections. (**a**) Boxplot of the thickness of thermohaline staircases picked manually from the CTD profiles. (**b**) Boxplot of the thickness of all thermohaline staircases picked from CT (left), calculated from the synthetic seismic from the CTD data (middle) and calculated from the real seismic section’s traces.

Quantitatively, we are interested in whether we can rely on seismic oceanography as an indirect method to assess the thickness of steps in thermohaline staircases and their spatial variability and coherence. To create a validation data set, we manually picked all the interfaces present in the CT profiles related to the steps of the thermohaline staircases (i.e., the salt finger layer top and the constant temperature and salinity layer top). The picking starts at 1000 m of water depth for eighteen CTDs (Fig. 7). Then, we calculate the thickness of each step for each available CTD profile, by computing the difference in depth between consecutive interfaces. A statistical analysis of these data shows that the median average thickness for the available CTD profiles is approximately 20 m. The results are consistent independently of the geographical location of the CTD profile, the depth and time of acquisition (i.e., season of the year) (Fig. 7a). Then, we estimated the thermohaline staircase steps’ thickness from the observed SO sections and the synthetic one computed along the CTD data (Fig. 7b). We use the wavelength of each individual seismic trace for the same depth interval (from 1.5 to 2 km) as a proxy of the true thickness. The average thickness estimated from the synthetic SO data agrees with those computed directly from the CTD profiles but has lower variability. This effect may be explained by the lower vertical resolution of the SO data compared with the CTD data and the tuning effect caused by the dominant frequency of the wavelet of the SO data. The thicknesses estimated from the observed SO sections do underestimate the actual thickness of the thermohaline staircases.

**Figure 8:**
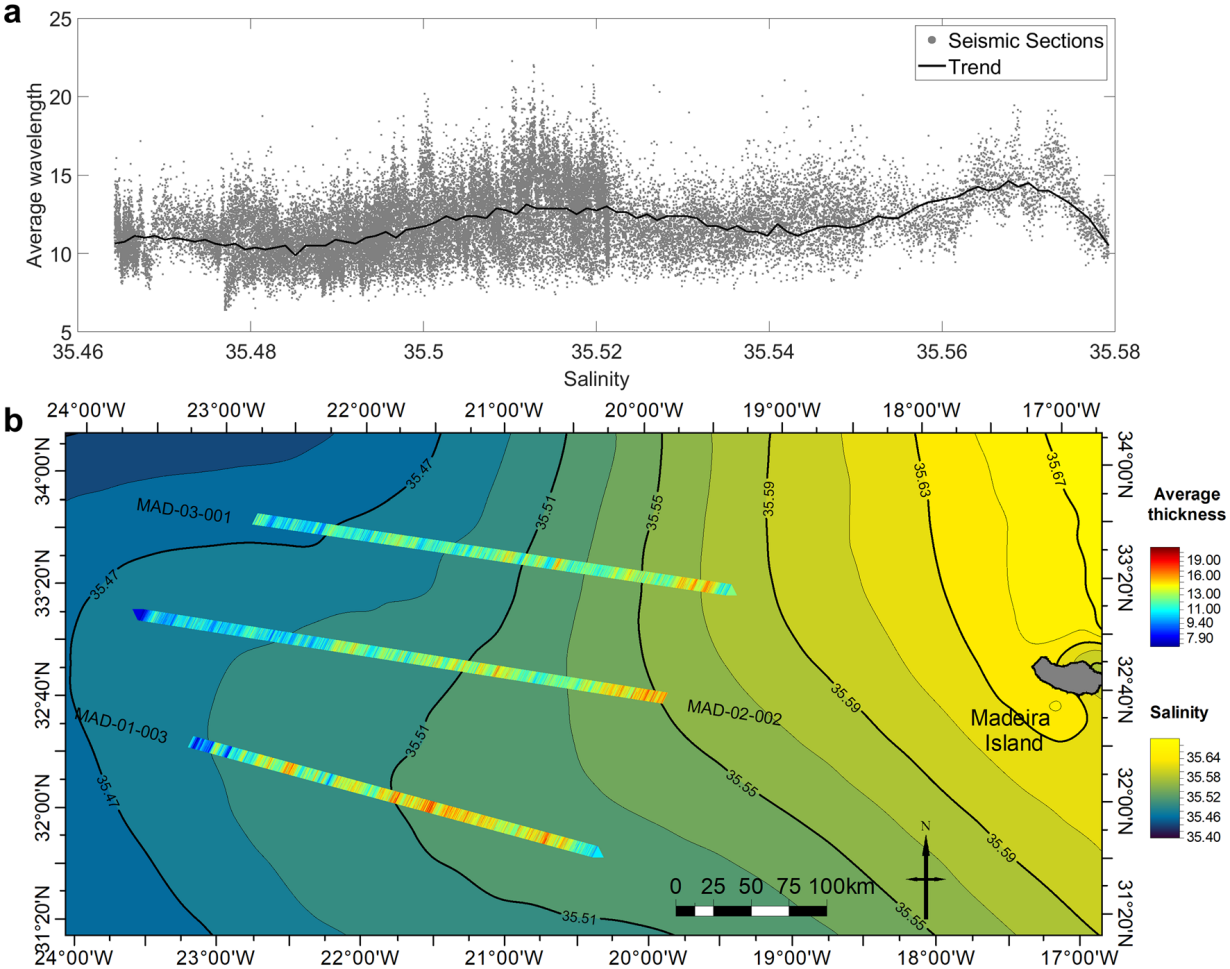
Comparison of thermohaline staircases thickness with regional salinity. (**a**) Scatterplot between the collocated salinity and average thermohaline staircases thickness for the three seismic sections (gray dots) and calculated trend (black line). (**b**) Map of the thickness of thermohaline staircases calculated from recorded seismic data on top of the mean salinity distribution for 1300 m of depth (salinity map extracted from the WOA18^30^).

Figure 8 shows the spatial variation of the thickness inferred along the SO sections for each seismic trace (i.e., vertical location). The results show that the thicknesses of the steps of the thermohaline staircases increase from West to East.

Using these results and the thickness data, we plotted the average of the estimated thicknesses for each seismic line (Fig. 8) over the salinity climatology at 1300 m extracted from the WOA18^30^. From Fig. 8a, it is possible to correlate the thickness increase of the steps of the thermohaline staircases with the salinity increase, the thickness and prevalence of staircases increase to the East. From Fig. 8b, we see that the thermohaline staircases tend to be more persistent and have higher thicknesses in places where salinity is also more constant. The average thickness computed from the seismic sections (Fig. 8) shows a relationship with the salinity gradient. Salinity decreases to the West of our study area as well as the mean thickness of the steps of the thermohaline staircases retrieved from the seismic sections.

## Discussion

Evidence of thermohaline staircases associated with the MOW have been reported since the 70s^3,7^. In an early paper, Elliot et al.^31^ found homogeneous layers in the subtropical Northeast Atlantic at several depths. However, the authors found that the step-layers around 1500 m, just beneath the MOW, to be recurrent in widely spaced observed stations. They suggested that these staircases could be coherent over distances of many tens of kilometers unlike the ones found on the upper layers (i.e., above the MOW). This spatial coherence was however hard to confirm with discrete and sparse T-S profiles obtained from CTD casts. In this study, we provide new evidence of the coherence of double-diffusive homogenous layers integrating direct and indirect observations of the ocean. We provide a coherent quantitative method to estimate the depth of thermohaline staircases from seismic oceanography data.

Coherent and quasi-permanent staircases in the MAP, similar to the ones that have been reported in other areas like the Tyrrhenian Sea^13^ and in the Caribbean Sea^15^. While there is still no consensus on what mechanisms drive the Ocean Meridional Overturning^11^, it is clear that the interior ocean mixing is one of its principal drivers and the interest of the physical oceanographic community on understanding the mixing processes has been renovated over the last decades.

The dynamics^2^, global distribution and relevance for ocean mixing^7^ of double-diffusive layers are still under debate, and the development of complementary methods for their study is highly valuable. In particular, seismic methods^20^ will be particularly useful to investigate their lateral structure and spatial coherence as well as their geographical extent due to the large amounts of multichannel seismic reflection data acquired in most margins.

## Conclusions

The ocean dynamics in the Madeira Abyssal Plain are still poorly understood due to the lack of spatially exhaustive sampling of the water column in the region of the globe. We combine direct observations of the water column and indirect measurements, as represented by seismic oceanography data, to quantitatively describe the presence of stable spatial and temporal thermohaline staircases in the region. The observed seismic signatures are validated by contrasting against numerical examples and field observations and have the potential to increase the current understanding about thermohaline staircases. While the proposed method is illustrated in this area, similar approaches might be applied to other oceanic settings leveraging the wide spread of multichannel seismic reflection data in most oceanic basins worldwide.

## Methodology

This section summarizes the methodological workflow adopted in this work. We start by gathering CTD data and processing the available temperature and salinity profiles to obtain the conservative temperature (CT) and absolute salinity (AS) profiles for each of the 18 CTD locations within the area of interest (Fig. 1). After processing, we computed the corresponding water density and sound speed (i.e., P-wave propagation velocity). These profiles were used to calculate the normal incidence reflection coefficients, which were then used to produce numerically synthetic seismic reflection data. The processed CTD data and the synthetic seismic traces were used to validate the origin of seismic reflections identified in the processed 2-D multichannel seismic reflection sections.

### Seismic oceanography data processing

Data processing is a critical step in SO. A suitable processing workflow allows obtaining a reliable and high-quality image of the ocean water structure. The seismic processing workflow applied to the available multichannel seismic sections differs from conventional seismic processing by recovering and enhancing weak seismic reflections from acoustic contrasts due to water temperature and salinity changes. The processing workflow represents a challenge not only due to the weak amplitudes of the reflected signal but also the effect of the direct wave (i.e., the energy that travels directly from the acoustic source to the receivers without being reflected or refracted) that is often the strongest and most easily identified signal in the seismic data. This seismic event can obscure the important reflections that contain information about the structure from the first few meters below the sea surface, which might be important to describe the ocean–atmosphere interaction.

The SO processing workflow applied to the SO data set has two main objectives. The first is to attenuate the direct arrival effect. The second is to preserve the relative amplitudes of the water reflections to be later used in quantitative studies (i.e., seismic inversion)^5,32^. To reach these objectives, the raw data was processed to remove low signal-to-noise ratio seismic traces, apply the acquisition geometry definition, filter noise, attenuate the direct arrival using a combination of linear move out, horizontal median filtering and amplitude subtraction and to correct for the spherical divergence. The processing continues with a common mid-point (CMP) sorting, which gathers seismic traces that share a common midpoint (i.e., the location at the subsurface where multiple reflections happen). The CMP gathers were corrected for normal move out (NMO), after a detailed velocity analysis was performed. NMO corrects for the time-dependency between source and receiver. The corrected gathers were then stacked. The stacking results in a single trace per CMP location and greatly increases the signal-to-noise ratio of the seismic image. The last processing step is the migration, which given a velocity model, corrects spatially the location of the seismic reflections and increase the horizontal resolution to approximately the CMP interval. The vertical domain of each SO section is in two-way travel time (TWT) and not in depth. We converted the TWT vertical domain into depth using the velocity model mentioned above.

The processed 2-D SO sections depict the vertical structure of the water column. As an illustrative example, we show the section MAD-01-003 after migration (Supplementary Fig. S1). A preliminary interpretation allows vertically dividing the SO section into three main parts: the top of the water column until ~ 3000 m comprises the zone with variable amplitude content, where the reflections are generated by fast variations in sound speed and density (Supplementary Fig. S1a); the bottom of the water column, from where there are no visible reflections until it reaches the seafloor (Supplementary Fig. S1b), implying that the water on those depths is fairly well mixed and therefore the acoustic energy did not encounter an interface to be reflected. The last zone of the section corresponds to the image of the seafloor and sediments beneath (Supplementary Fig. S1c). The remaining 2-D SO sections exhibit a similar behavior.

### Direct measurements calculations

The CTD profiles were used to calculate synthetic seismic traces and to identify regions where thermohaline staircases happen. Synthetic seismic traces were computed with the international thermodynamic equations of seawater^28^ available in the TEOS-10 software v3.06 in MATLAB^®^. To calculate water density and sound speed from the CTD profiles we used CT and AS, corrected from in-situ measurements using the TEOS^28,33,34^. CTD data were then used to compute synthetic seismic response along the CTD profiles. First, we calculated the density $(\rho )$^34^ and sound speed $({V}_{p})$^35^ using the TEOS. Then P-impedance (${I}_{P}$) profiles (Eq. 1) were computed following

1 $${I}_{P}=\rho \times {v}_{p}[{\text{kg}}/{{\text{m}}}^{2}\mathrm{ s}]$$

The normal-incidence acoustic reflection coefficient ($$RC)$$ profiles were computed following:

2 $$RC=\frac{{IP}_{i+1}-{IP}_{i}}{{IP}_{i+1}+{IP}_{i}},$$

where $i$ is the sample above the interface, where $RC$ is being computed, and $i+1$ is the sample below. Finally, the synthetic seismic response ($s$) is obtained by the convolution of the $RC$ with a wavelet ($w$) (Eq. 3). The wavelet can be considered a numerical representation of the acoustic wave field generated at the source location and propagated within the system under investigation (i.e., the water column):

3 $$s=RC\times w.$$

The CTD data were also used to infer whether the area has physical conditions to form thermohaline staircases. To assess this possibility the Brunt–Väisälä frequency squared $\left({N}^{2}\right)$^34–36^ (i.e., a way to determine how resistant a fluid is to vertical movements), the Turner angle ($TU$), and the density ratio ($R\rho$), to determine the origin and intensity of the double-diffusive regime, were computed in each profile using the TEOS.

The vertical variation of $TU$ provides a good stratification pattern for the unstable diffusion of salt-fingers. This oceanographic phenomenon can be interpreted with $TU$ comprehended between 45° and 90°. The following set of equations defines $TU$ in degrees, with $\alpha =-{\rho }^{-1}(\partial\uprho /\partial {\text{CT}})$ the coefficient of thermal expansion and $\beta =-{\rho }^{-1}(\partial\uprho /\partial {\text{SA}})$ the coefficient of saline contraction:

4 $$TU={\mathit{tan}}^{-1}\left(\alpha \frac{\partial CT}{\partial z}+\beta \frac{\partial SA}{\partial z},\alpha \frac{\partial CT}{\partial z}-\beta \frac{\partial SA}{\partial z}\right).$$

The $R\rho$, which can be calculated from the $TU$ (Eq. 4), gives the condition for the formation of staircases such as 1<$R\rho$  <  ~ 2^37^.

5 $$R\rho =\frac{\alpha \Delta CT}{\beta \Delta SA}.$$

The Brunt–Väisälä frequency analysis also discriminates the layers with high frequency as suffering from high mixing gradients:

6 $${N}^{2}=-\frac{g}{{\rho }_{0}}\frac{\partial \rho \left(z\right)}{\partial z}=g\left(\alpha \frac{\partial CT}{\partial z}-\beta \frac{\partial SA}{\partial z}\right).$$

With $\rho$ as density in function of depth. These layers are prone to double diffusion regimes and low buoyancy frequency from well-mixed layers and not stratified^38^. These three parameters give us the ideal conditions for the step-like formations from salt fingering (Supplementary Fig. S2).

### Supplementary Information

Supplementary Information 1.

Supplementary Information 2.

Supplementary Information 3.
