# Supplementary figures and images for: Acoustic imaging of stable double diffusion in the Madeira abyssal plain
Source: Sci Rep. 2024 Apr 9;14:8273. doi: 10.1038/s41598-024-58861-7 (PMC11004020; doi:10.1038/s41598-024-58861-7)

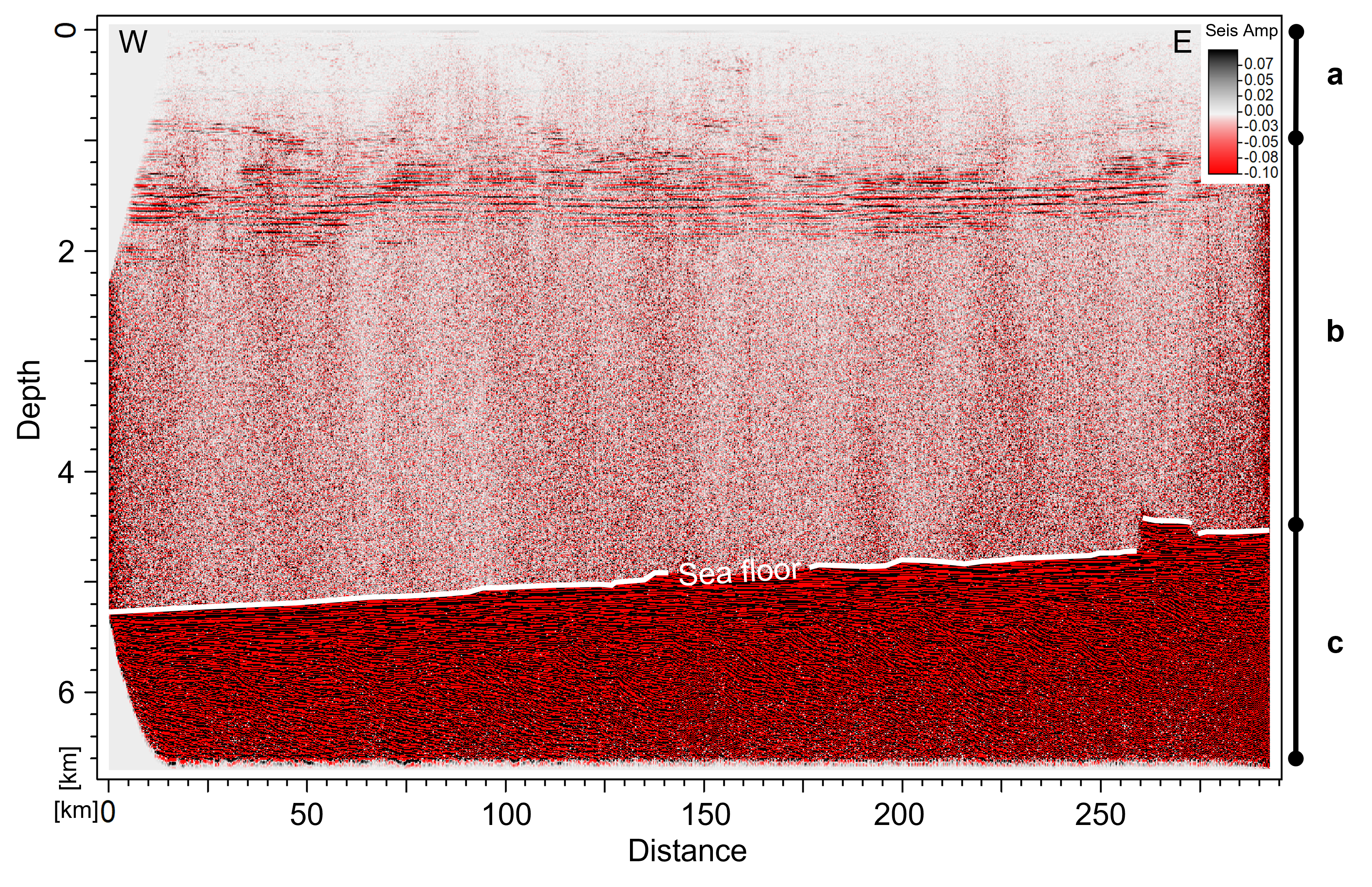

Supplement: Supplementary file 1 — Supplementary Information 1. [file 41598_2024_58861_MOESM1_ESM.tif]

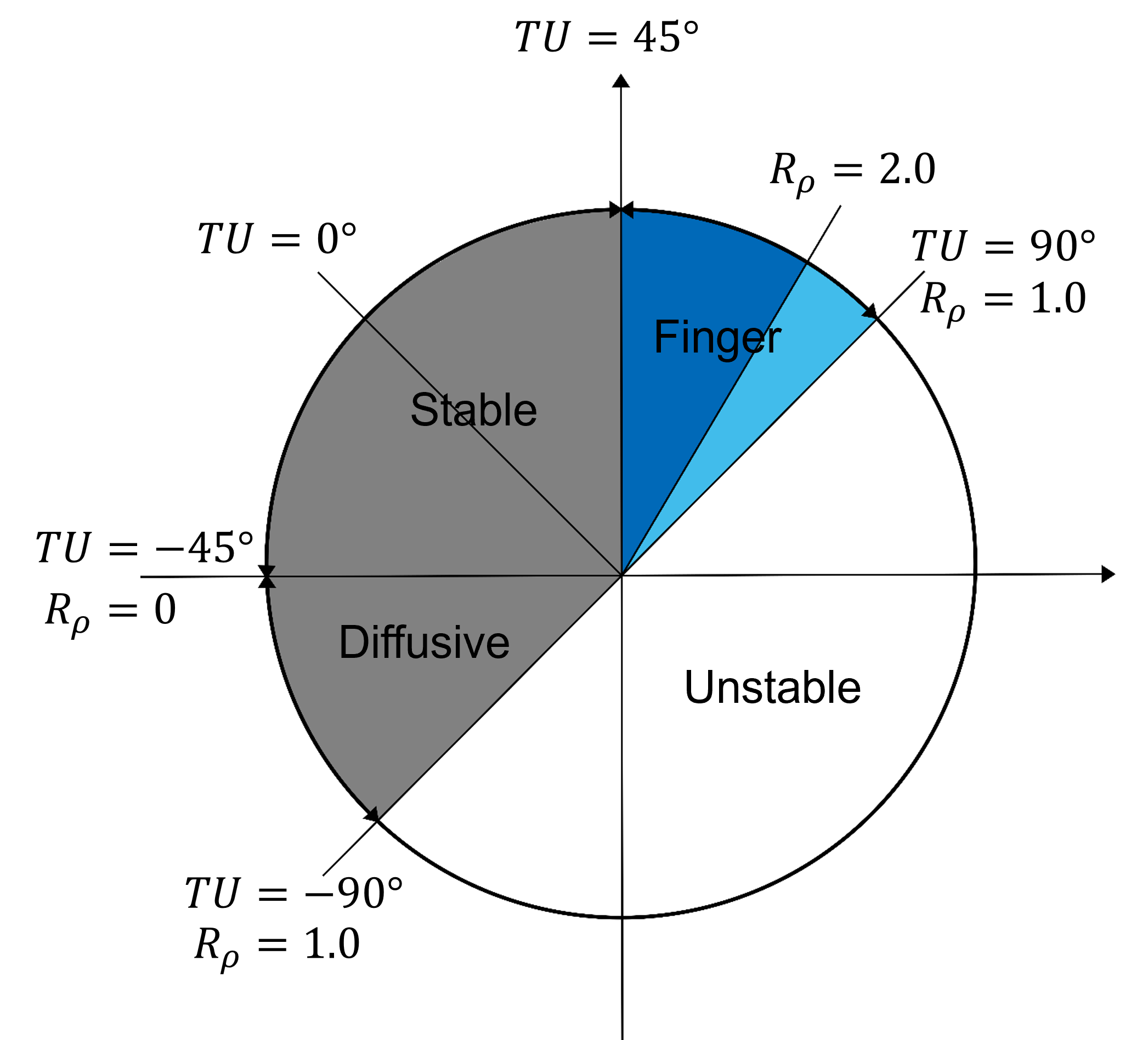

Supplement: Supplementary file 2 — Supplementary Information 2. [file 41598_2024_58861_MOESM2_ESM.tif]
