# Supplementary material for: Acoustic imaging of stable double diffusion in the Madeira abyssal plain
Source: Sci Rep. 2024 Apr 9;14:8273. doi: 10.1038/s41598-024-58861-7 (PMC11004020; doi:10.1038/s41598-024-58861-7)
Supplement: Supplementary file 3 — Supplementary Information 3. [file 41598_2024_58861_MOESM3_ESM.docx]

Fig. S1: Final processed seismic section (MAD-01-003) cropped at 9 seconds TWT. Top (**a**) (until 3 km depth) comprises reflections from the water column; middle (**b**) (up to seafloor) is reflection free; bottom (**c**), encompasses the seafloor and subsurface sediment bellow.

Fig. S2: Schematic representation of the definition of the Turner angle (°), and regions of instability due to double-diffusion (modified from You^7^).
